# Evaluation of the five-year operation period of a rapid response team led by an intensive care physician at a university hospital

**DOI:** 10.5935/0103-507X.20160045

**Published:** 2016

**Authors:** Ana Luiza Mezzaroba, Marcos Toshiyuki Tanita, Josiane Festti, Claudia Maria Dantas de Maio Carrilho, Lucienne Tibery Queiroz Cardoso, Cintia Magalhães Carvalho Grion

**Affiliations:** 1 Universidade Estadual de Londrina - Londrina (PR), Brazil.

**Keywords:** Hospital rapid response team, Hospital mortality, Hospital, universities, Patient safety, Intensive care units

## Abstract

**Objective:**

To evaluate the implementation of a multidisciplinary rapid response team led by an intensive care physician at a university hospital.

**Methods:**

This retrospective cohort study analyzed assessment forms that were completed during the assessments made by the rapid response team of a university hospital between March 2009 and February 2014.

**Results:**

Data were collected from 1,628 assessments performed by the rapid response team for 1,024 patients and included 1,423 code yellow events and 205 code blue events. The number of assessments was higher in the first year of operation of the rapid response team. The multivariate analysis indicated that age (OR 1.02; 95%CI 1.02 - 1.03; p < 0.001), being male (OR 1.48; 95%CI 1.09 - 2.01; p = 0.01), having more than one assessment (OR 3.31; 95%CI, 2.32 - 4.71; p < 0.001), hospitalization for clinical care (OR 1.77; 95%CI 1.29 - 2.42; p < 0.001), the request of admission to the intensive care unit after the code event (OR 4.75; 95%CI 3.43 - 6.59; p < 0.001), and admission to the intensive care unit before the code event (OR 2.13; 95%CI 1.41 - 3.21; p = 0.001) were risk factors for hospital mortality in patients who were seen for code yellow events.

**Conclusion:**

The hospital mortality rates were higher than those found in previous studies. The number of assessments was higher in the first year of operation of the rapid response team. Moreover, hospital mortality was higher among patients admitted for clinical care.

## Abstract (pt)

**Objetivo:**

Avaliar a implementação de time de resposta rápida multidisciplinar liderado por médico intensivista em hospital universitário.

**Métodos:**

Estudo de coorte retrospectiva realizado pela análise de fichas de atendimentos preenchidas durante os atendimentos realizados pelo time de resposta rápida do hospital universitário entre março de 2009 e fevereiro de 2014.

**Resultados:**

Foram coletados dados de 1.628 atendimentos realizados em 1.024 pacientes pelo time de resposta rápida, sendo 1.423 códigos amarelos e 205 códigos azuis. Houve maior número de atendimentos no primeiro ano, após implementação do time de resposta rápida. A análise multivariada identificou idade (OR 1,02; IC95% 1,02 - 1,03; p < 0,001), sexo masculino (OR 1,48; IC95% 1,09 - 2,01; p = 0,01), mais de um atendimento (OR 3,31; IC95% 2,32 - 4,71; p < 0,001), internação para especialidades clínicas (OR 1,77; IC95% 1,29 - 2,42; p < 0,001), pedido de vaga de unidade de terapia intensiva posterior ao código (OR 4,75; IC95% 3,43 - 6,59; p < 0,001) e admissão em unidade de terapia intensiva prévia ao código (OR 2,13, IC95% 1,41 - 3,21; p = 0,001) como fatores de risco para mortalidade hospitalar de pacientes atendidos em códigos amarelos.

**Conclusão:**

Os índices de mortalidade hospitalar foram elevados quando comparados aos da literatura e houve maior número de atendimentos no primeiro ano de atuação do time de resposta rápida. Houve maior mortalidade hospitalar entre pacientes internados para especialidades clínicas.

## INTRODUCTION

The increased complexity of hospitalized patients has led to an increase in the number of adverse events, despite technological advancements and the development of new drugs.^(1)^ Adverse events are defined as any threat to the life of patients under medical treatment; these events may be the result of errors and are associated with higher rates of complications and increased mortality. Cardiac arrest outside the monitored environment of intensive care units is considered a serious adverse event that can potentially be avoided.^(2)^

Several studies have shown that early warning signs typically occur six to eight hours before most cases of cardiac arrest in hospitalized patients.^(1,3,4)^ Therefore, there is a window of time that can be used to identify patients at risk of death and to implement appropriate interventions. An appropriate strategy should involve the proper measurement and recording of vital signs and the establishment of abnormality thresholds.^(5,6)^ In theory, the early identification of these signs and appropriate treatment should improve the survival of hospitalized patients.^(7)^

Rapid response systems (RRS) are intended to increase patient safety during hospitalization, and the decreased number of cardiac arrests outside the intensive care setting is an indicator of quality.^(8,9)^ However, the results of studies that evaluate the effectiveness of RRS are conflicting. Although a large multicenter study fails to demonstrate a decrease in hospital mortality after the implementation of RRS,^(6)^ the validity of smaller studies with contrasting results should also be considered.^(10-15)^ In addition, RRS may help promote the continuous training of staff members to handle emergencies and improve the safety of hospitalized patients.^(16)^

The aim of this study is to evaluate the implementation of a multidisciplinary rapid response team led by an intensive care physician at a tertiary university hospital.

## METHODS

This retrospective cohort study was conducted between March 2009 and February 2014. It analyzed data from assessment forms that were completed by the nurses and physicians of a rapid response team (RRT) at the time of activation of codes yellow and blue. Additional clinical data from patients were collected from the computerized system of the *Hospital Universitário* (HU) of the *Universidade Estadual de Londrina* (UEL). The number of hospitalizations in the hospital sectors serviced by the RRT between 2009 and 2014 was provided by the Statistics Section of the Division of Medical Records and Statistics of HU-UEL. This study was approved by the local Research Ethics Committee under Protocol No. 547.204, and the requirement of a signed informed consent form was waived. The HU is a supplementary body of UEL and is the largest general public hospital serving the city of Londrina and neighboring cities in the state of Paraná. It had 315 beds during the study period.

Requests for assessment by the RRT were based on the following criteria: cardiorespiratory arrest (code blue) and clinical instability data (code yellow), including peripheral oxygen saturation lower than 90%, a respiratory rate lower than 10 bpm or higher than 30bpm, systolic blood pressure lower than 90mmHg, systolic blood pressure higher than 180mmHg with symptoms, a heart rate lower than 45bpm or higher than 125bpm, a decreased level of consciousness, seizures, or serious concerns of the medical team with regard to the general clinical status of the patient. The time goal for the arrival of the intensive care physician from the RRT to the point of care was two minutes for code blue events and five minutes for code yellow events.

The RRT working at the HU-UEL was composed of an intensive care physician and physical therapist and was involved in handling the requests for treatment of code events in the adult patient wards. The RRT started operating in March 2009, and because of human resource limitations, the team worked 12 hours a day, from 7:00 am to 7:00 p.m., every day of the week. At night, requests were handled by the staff on duty in the emergency departments; however, these services were not included in the analysis. Another function of the RRT was the performance of daily assessments (in the morning and afternoon) of critically ill patients who were not admitted to the intensive care unit (ICU); this was necessary because of the presence of patients with indication of admission to the ICU who did not obtain immediate access to this sector because of the unavailability of beds. In these cases, the RRT improved patient safety by making daily physical examinations, reviewing medical prescriptions, checking the test results, and guiding the professionals who were responsible for the care of these patients. This activity was performed throughout the period that the patient waited for a vacancy until his/her transfer to the ICU or until the referral was canceled owing to clinical improvement or transfer.

The study population included hospitalized critically ill patients with an indication for medical assessment, treatment, and follow-up by the RRT. All assessments made by the RRT in the study period were included. Patients younger than 18 years of age and those whose assessment records had insufficient data were excluded.

Data from patients enrolled in the study were collected up until the hospital outcome. Clinical, demographic, and treatment data were collected at patient enrollment. These data included age, gender, the type of medical care provided (clinical or surgical), the site of the adverse events, and the time of identification of changes in patient status, in addition to the time of activation of the RRT, the time of arrival of the RRT, the time of assessment by the RRT, the reasons for activating the RRT, the diagnosis at the time of hospitalization, the diagnosis made by the RRT, the interventions made by the RRT, the request for admission at the ICU after assessment by the RRT, the date and diagnosis at admission to the ICU, survival at hospital discharge, and transfer to palliative care.

The results of the continuous variables were expressed as the mean and standard deviation (SD), or median and interquartile ranges (ITQ), according to data distribution. Student's *t*-test was used to compare the means of continuous variables with normal distribution and variance homogeneity. The Mann-Whitney nonparametric test was used to compare data with non-normal distribution and/or variance heterogeneity. Categorical data were expressed as frequencies and analyzed using the chi-square test. Simple and multiple regression analyses were conducted to estimate the prediction model of hospital outcome, together with the forward stepwise selection method of the variables; p values lower than 0.20 were used as the criterion for inclusion in the model, and p values lower than 0.05 were used as the criterion for remaining in the model. Hospital mortality data were analyzed using the Kaplan-Meier survival curve and reported as frequencies. Statistical analyses were performed using the MedCalc statistical software version 15.2.2 (MedCalc Software bvba, Ostend, Belgium) at a level of significance of 5%.

## RESULTS

A total of 1,674 code-based assessments were performed during the study period. Five code blue forms and 41 code yellow forms were excluded because of insufficient data. Therefore, 1,628 assessments were analyzed, of which 1,423 requests involved yellow code events (87.4%) and 205 requests involved code blue events (12.6%). It is noteworthy that only 1,024 patients were seen because more than one assessment was requested for some patients. Among the 1,024 patients seen, 844 were seen for code yellow events only, 99 were seen for code yellow and code blue events, and 81 were seen for code blue events only. The analysis of the number of admissions in inpatient units where the RRT operated allowed the number of code yellow and code blue events per thousand hospitalizations during the study period to be calculated (Table 1).

**Table 1:** Number of code yellow and code blue events

	Code yellow events per 1,000 admissions	OR (95%CI)*	Code blue events per 1,000 admissions	OR (95%CI)*
Year 1	102.15	1	12.91	1
Year 2	42.1	0.39 (0.33 - 0.46)	4.54	0.35 (0.22 - 0.56)
Year 3	37.78	0.35 (0.29 - 0.41)	6.55	0.50 (0.33 - 0.76)
Year 4	44.8	0.41 (0.35 - 0.48)	6.74	0.52 (0.35 - 0.78)
Year 5	49.12	0.45 (0.39 - 0.53)	8.93	0.69 (0.47 - 1.01)

OR - odds ratio; 95%CI - 95% confidence interval; Year 1: March 2009 to February 2010; Year 2: March 2010 to February 2011; Year 3: March 2011 to February 2012; Year 4: March 2012 to February 2013; Year 5: March 2013 to February 2014.

* p < 0.001 using a chi-square test for the overall trend.

The average number of assessments per patient was 1.50 ± 1.00 among the 943 patients seen for code yellow events and 1.13 ± 0.41 among the 180 patients seen for code blue events. The clinical characteristics of the patients and the variables related to the assessments for code yellow and code blue events are described in table 2.

**Table 2:** Characteristics of the patients seen for code yellow and code blue events

	Code yellow events	Code blue events
Age (years)	61.9 (18.19)	63.02 (17.66)
Male gender	54.9	51.2
Diagnosis at admission		
CPAD/AAO	11.4	11.7
Fractures	9.8	12.2
S/SAH	9.2	15.1
EL/PSC	5.9	4.9
Pneumonia	5.4	2.4
Hematologic cancers	2.6	4.9
Others	55.7	48.8
Surgical care	56	57.6
Period 1 (minutes)	1 (0 - 5)	0 (0 - 1)
Period 2 (minutes)	2 (1 - 3)	1 (0 - 2)
Period 3 (minutes)	33 (19 - 57)	29 (15 - 45)
Transfer to the ICU	27.1	5.4
Transfer to palliative care	5.3	7.8

CPAD/AAO - chronic peripheral arterial disease/acute arterial occlusion; S/SAH - stroke/subarachnoid hemorrhage; EL/ES - exploratory laparotomy/emergency surgery; ICU - intensive care unit. Period 1: the period from the detection of changes in clinical status to activation of the rapid response team; Period 2: time taken for the arrival of the rapid response team; Period 3: time taken for the assessment. The results are shown as mean (standard deviation), percentage, or median (interquartile range).

The reasons for the requests related to code yellow events are listed in table 3, and each assessment form could contain more than one reason. The activities developed by the RRT for addressing code yellow events were divided into guidelines, procedures, treatments, and tests requested (Table 4).

**Table 3:** Reasons for the activation of 1,423 code yellow events

Reasons	N (%)
Hospital team was seriously concerned about the patient	536 (37.7)
Peripheral oxygen saturation lower than 90%	459 (32.3)
Changes in respiratory rate	398 (28.0)
Systolic blood pressure lower than 90 mmHg	383 (26.9)
Decreased level of consciousness	358 (25.2)
Changes in heart rate	231 (16.2)
Seizures	98 (6.9)
Systolic blood pressure higher than 180 mmHg	50 (3.5)

**Table 4:** Activities developed during assessment of 1,423 code yellow events

Activities	N (%)
Guidelines	
Call the physician responsible for defining individual therapy	112 (7.9)
Request a physical therapist	51 (3.6)
Insertion of central venous access	42 (3.0)
Discussion of the limitations of the therapeutic support	17 (1.2)
Indication of surgical approaches	3 (0.2)
Other	202 (14.2)
Procedures	
Return the patient to mechanical ventilation	312 (21.9)
Endotracheal intubation	245 (17.2)
Insertion of central venous catheter	93 (6.5)
Aspiration via oral cavity, endotracheal tube, or tracheostomy	72 (5.1)
Use of a Sengstaken-Blakemore balloon	3 (0.2)
Other	47 (3.3)
Treatments	
Volume prescription	395 (27.8)
Vasoactive drugs	362 (25.4)
Antibiotics	265 (18.6)
Sedation	211 (14.8)
Other	751 (52.8)
Tests	
Hematological and biochemical tests	370 (26.0)
Chest radiography	222 (15.6)
Blood culture, urine culture, or tracheal aspirate culture	168 (11.8)
Electrocardiography	165 (11.6)
Computed tomography	63 (4.4)
Other	28 (2.0)

The hospital mortality of patients who required assessment for code yellow events during hospitalization was 67.7%; after excluding patients in palliative care, the hospital mortality of patients assessed for code yellow events was 66%.

The need for multiple assessments was more frequent in surgical patients compared to clinical patients for both code yellow and code blue events. Of the patients who required multiple assessments for code yellow events, 57.3% were admitted for surgical care. Among those who needed multiple assessments for code blue events, 66.6% were admitted for surgical care. It should be considered that 88.5% of admissions were classified as surgical after adult patients who were admitted to the sectors serviced by the RRT during the study period were evaluated. However, when all code events were analyzed, mortality was lower after the assessment of cases considered surgical (68.63%) compared to cases considered clinical (75.60%, p = 0.001).

**Figure 1:**
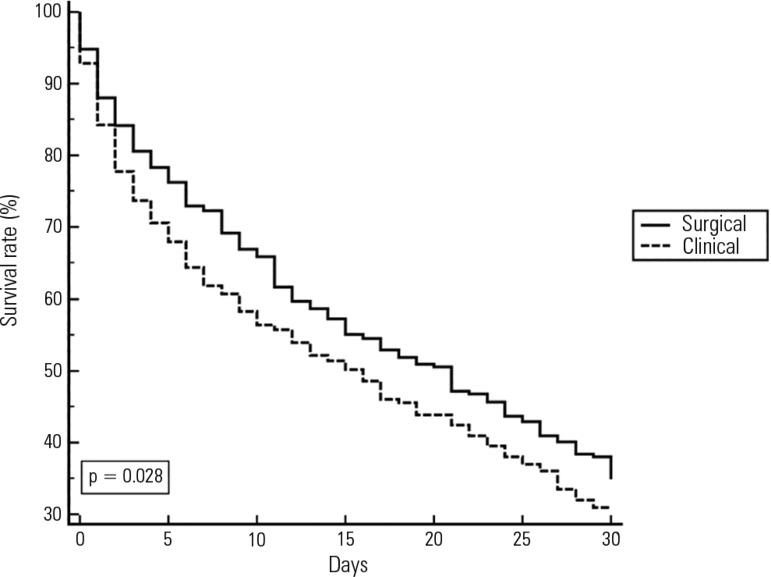
Kaplan-Meier survival curve for clinical and surgical patients assessed for code yellow events at day 30 after the first code yellow event.

For code yellow events, mortality was lower among patients with single assessments (53.6%) compared to those with multiple assessments (80.0%; p < 0.001). The univariate and multivariate analyses indicated that the risk factors that remained in the model for mortality in the patients seen for code yellow events were being male, age in years, the need for multiple assessments, hospitalization for clinical care, the request for admission to the ICU after the code event, and staying in the ICU before the code event in the same hospitalization (Table 5). Moreover, the analysis of the Kaplan-Meier curve (Figure 1) indicated a lower survival rate at 30 days for patients who were hospitalized for clinical care and seen for code yellow events, counted from the day when the first code yellow event was assessed.

**Table 5:** Univariate and multivariate analyses of the risk factors for mortality of patients seen by the rapid response team for code yellow events

Variables	Univariate			Multivariate*		
	OR	95%CI	p-value	OR	95%CI	p-value
Age (years)	1.02	1.02 - 1.03	< 0.001	1.02	1.02 - 1.03	< 0.001
Male gender	1.48	1.9 - 2.1	0.01	1.48	1.09 - 2.01	0.01
More than one code event	3.31	2.32 - 4.71	< 0.001	3.31	2.32 - 4.71	< 0.001
Clinical patients	1.77	1.29 - 2.42	< 0.001	1.77	1.29 - 2.42	< 0.001
Time (min)†	0.99	0.99 - 1.00	0.91			
Request for admission to the ICU‡	4.75	3.43 - 6.59	< 0.001	4.75	3.43 - 6.59	< 0.001
Admission to the ICU before the code event§	2.13	1.41 - 3.21	< 0.001	2.13	1.41 - 3.21	< 0.001

OR, odds ratio; 95% CI, 95% confidence interval; ICU, intensive care unit;

* Logistic regression analysis using the forward stepwise method;

† Time between the diagnosis of changes in the clinical status and the activation of the rapid response team;

‡ Request for admission to the ICU after the code event;

§ Request for admission to the ICU before the code event (patients previously admitted to the ICU in the same hospitalization).

## DISCUSSION

This study evaluated the clinical and epidemiological profile of the assessments performed by an RRT led by an intensive care physician at a tertiary university hospital over the course of five years.

Other studies have demonstrated a direct relationship between the time of operation of the RRT and improvements in quality indicators, leading to increased safety of the hospitalized patients.^(17,18)^ The RRT assessed in this study did not operate full-time at the study site, as opposed to what is more commonly reported in the literature. The decision to implement an RRT with partial activity was made because there was not sufficient financial resources available for the implementation of the full service. Therefore, our results are compared to those of studies that included an RRT with full-time activity, and for this reason, data should be interpreted considering this fact. Although the RRT did not operate full-time during the study period, a high number of assessments, which was higher than the average number reported in the literature,^(10-13)^ were performed. The number of assessments for code yellow and code blue events was higher in the first year of operation of the RRT, and many patients needed to be assessed for more than one code event. In addition, requests for the assessment of patients who were admitted for surgical care predominated; however, the mortality of patients who were admitted for clinical care was higher. In addition, hospital mortality rates, which are the final assessment outcome in this study, were higher than those reported in the literature even when patients in palliative care were not considered.^(10,14)^

The results of this study are not consistent with the usual trend in the number of requests for assessments by the RRT seen in the literature, which usually reveals a gradual increase in the number of requests over time.^(15,19-22)^ In general, previous studies have shown a certain preliminary resistance to the implementation of an RRT for several reasons, including the habit of calling the physician who is responsible for the patient to address existing complications, a lack of knowledge about the RRT, disagreement with the criteria adopted for activating the RRT, or apprehension about incorrectly activating the RRT in the face of code events.^(20)^

In this study, the number of assessments was higher in the first year of operation of the RRT compared to the following years, most likely because of the pre-existing need for inclusion of an intensive care physician in the care of hospitalized patients who were not admitted to the ICU. In the research institution, owing to the unavailability of ICU beds, the health care team working in inpatient units frequently provided care to critically ill patients. In the year of implementation of the RRT, there was a wide dissemination of this new care service, resulting in a large number of service requests. The entire team may have been inexperienced in the first year of operation of this team, resulting in unnecessary requests for code events and increasing staff surveillance for warning signs. Furthermore, the subsequent decrease in the number of code events recorded in the assessment forms may be correlated with the underreporting of code events, forgetfulness, overwork, and a lack of continuous training for the hospital staff and RRT instead of an actual decrease in the number of services. These factors indicate the inexperience of the RRT in providing the service evaluated, most likely because the hospital was a public and teaching institution, with a high turnover of staff and students.

However, the decrease in the number of code yellow events can also be interpreted as an optimization in the organization and logistics of care in inpatient units. The structuring of the services provided by the RRT, with the establishment of routine visits to critically ill hospitalized patients, may have contributed to the increased feeling of safety by the inpatient unit teams because of the increased presence of the intensive care physician, with a consequent decrease in the number of service requests.

Although the assessment records show a low number of discussions on palliative care provision between the medical specialists and the RRT, we believe that this role of the RRT is important. The evaluation of individual reports indicated that the discussions on palliative care were even less evident in the period before the implementation of the RRT in HU-UEL. In this context, the provision of care to critically ill patients in inpatient units by an intensive care physician has improved the approach to and the discussions on this topic with the specialists treating the patients and patient's families.

Hospital mortality in our patients was higher than that found in previous studies;^(10,15)^ this finding may be due to the following reasons: the hospital's status as a tertiary and reference center for complex cases; the existence of structural and overcapacity problems, which contribute to the high rate of hospital infections; and limited financial and human resources. The delay in admission of critically ill patients to the ICU owing to the lack of availability of beds should also be considered because there is evidence that each hour of delay in ICU admission increases hospital mortality.^(23)^

The decrease in the absolute and relative number of code blue events in the second year after the implementation of the RRT in our service suggests improvements in the safety of hospitalized patients. However, there was a concomitant decrease in the number of code yellow events in the same period, which was unexpected because previous studies have indicated a direct relationship between the RRT dose and its efficacy.^(24)^ This divergence can be explained by the performance of routine visits and the constant presence in inpatient units of an intensive care physician, who acted preventively, regardless of the number of code yellow events. Therefore, the RRT dose increased in the research institution, but this increase was not reflected in the number of code yellow events assessed. Additionally, the increase in the absolute and relative number of code blue events in year 5, compared to years 2, 3, and 4, underscores the need to improve the response of the medical team to warning signs and the occurrence of code yellow events before cardiac arrest.

This study has some limitations. First, its descriptive and single-center nature limits its external validity. Second, this study was based on the analysis of hospital records for the assessment of code events and is thus prone to errors and differences in record keeping. Third, the characteristics of the RRT system were different from other systems described in the literature, and therefore, the results of this study should be interpreted with caution. One of the strengths of our study is that it is one of the few Latin American studies that describes the operation of an RRT for an extended period and includes a large number of assessments.

## CONCLUSION

In this paper, we describe the epidemiological profile of patients with code yellow and blue events assessed by a rapid response team over a five-year period. The number of assessments for code yellow and code blue events was higher in the first year of operation of the rapid response team. Hospital mortality was higher for patients who were hospitalized for clinical care and for patients with multiple assessments.
